# Evidence-Based ZHENG: A Traditional Chinese Medicine Syndrome

**DOI:** 10.1155/2012/246538

**Published:** 2012-08-12

**Authors:** Shi-Bing Su, Aiping Lu, Shao Li, Wei Jia

**Affiliations:** ^1^Research Center for Complex System of Traditional Chinese Medicine, Shanghai University of Traditional Chinese Medicine, Shanghai 201203, China; ^2^Institute of Basic Research In Clinical Medicine, China Academy of Chinese Medical Sciences, Beijing 100700, China; ^3^MOE Key Laboratory of Bioinformatics and Bioinformatics Division, TNLIST/Department of Automation, Tsinghua University, Beijing 100084, China; ^4^Department of Nutrition, The University of North Carolina at Greensboro, Kannapolis, NC 28081, USA

The traditional Chinese medicine (TCM) ZHENG, also known as TCM syndrome or TCM pattern, is an integral and essential part of TCM theory. A TCM ZHENG is in essence a characteristic profile of all clinical manifestations that can be identified by a TCM practitioner. Clinical treatments of a patient rely on the successful differentiation of a specific ZHENG. Recent advances in systems biology have allowed the application of new phenotyping technologies in the study of the ZHENG differentiation with plausible biological interpretations. Understanding of the characteristic changes in biochemistry associated with a specific TCM ZHENG will facilitate the development of ZHENG identification and a novel disease diagnostic and stratification approach that will potentially lead to personalized healthcare strategies for a range of diseases that lack therapeutic solutions. Here, we have grouped together 28 excellent papers in this field and put forward for publication in this special issue on TCM ZHENG.

Firstly, there are 3 review or research papers in this special issue addressed the concept, origin, and development of ZHENG, the recent advances in ZHENG identification and its clinical applications, and latest technologies and methods such as omics methods and data mining for ZHENG identification and outcome measurement. Two papers reviewed the clinical characterization and molecular basis of TCM ZHENG in cancer and TCM management in hepatic encephalopathy, respectively. Moreover, a review paper reviewed systematically the classification of TCM ZHENGs associated with insomnia.

In TCM, the clinical diagnosis of ZHENG relies on the gathering of clinical information through inspection, auscultation and olfaction, inquiry, and palpation. For the acquisition of ZHENG-related clinical information, 2 research articles presented the established ZHENG questionnaire in the posthepatic cirrhosis and advanced cancer patients with constipation, respectively. Moreover, the patient stratification and personalized treatment by means of the ZHENG identification and approaches in patients with allergic rhinitis and subhealthy people with fatigue were presented. Additionally, the correlation between Blood-stasis syndrome score and cardio-ankle vascular index in stroke patients was also discussed.

ZHENG is not merely an assembly of many disease symptoms but an organization of interrelated clinical manifestations following the TCM theories. The interrelated symptoms and signs of diseases in the ZHENG measurement should be analyzed using appropriate statistical tools to better understand the ZHENG classification. Six research articles of this special issue presented the data mining of ZHENG differentiation using the combination of wavelet packet transform and sample entropy, the clinical phenotypic network in angina pectoris of coronary heart disease, a multilabel learning using the relevant feature for each label algorithm in chronic gastritis, and a structural equation modeling approach in suboptimal health status.

To objectively differentiate ZHENGs, 7 research articles in this issue presented the ZHENG classification using genes, proteins, metabolites, and/or their profiles. These are system strategies in investigating ZHENG classification and treatment evaluation by means of gene polymorphism, transcriptomics, proteomics, metabonomics, bioinformatics, and network pharmacology. The methods include IL-10genotypes in ZHENG, the metabonomic evaluation of ZHENG classification and treatment by Chinese herbal formula, and a combined ZHENG theory and high-throughout gene chip data to predict new effects of the formula in hepatitis B-caused cirrhosis. Additionally, the molecular mechanisms of “Same ZHENG for Different Diseases” and “Different ZHENGs for Same Disease” in chronic hepatitis B and liver cirrhosis and the ZHENG classification in chronic hepatitis B patients by SELDI-based protein chip analysis were also discussed in these papers.

To experimentally evaluate ZHENG, various pharmacological models of ZHENG are to be established. In this special issue, 4 papers discussed the establishment and/or the application of ZHENG animal models, encompassing the preparation of blood-deficient syndrome model in chicken, the castration-induced kidney deficiency syndrome model in arthritic rats, the kidney-yang deficiency syndrome model in Alzheimer's disease rats, and their applications. Moreover, it was also presented that Chinese herbal medicines were used to treat the established mouse xenograft pancreatic cancer models with dampness-heat, spleen-deficiency and Blood-stasis syndromes. 

In summary, the concept of TCM ZHENG, as a diagnostic approach in TCM, would provide invaluable guidance about the therapeutic choices and personalized disease management, not only in traditional medical practices but in modern healthcare systems as well. We look forward to an increasing number and sizes of clinical trials utilizing TCM ZHENG that will be conducted in the future to further promote the development of evidence-based personalized medicine.

*Shi-Bing Su* *Shi-Bing Su* *Aiping Lu* *Aiping Lu* *Shao Li* *Shao Li* *Wei Jia* *Wei Jia*

